# Conchal Cartilage in Surgical Reconstruction of Orbital Floor Fracture

**DOI:** 10.7759/cureus.13223

**Published:** 2021-02-08

**Authors:** Jayapaul Vaanmugil, Samson Jimson, Lokesh Bhanumurthy, M Arunprakash, Raveendharan Kandasamy

**Affiliations:** 1 Oral and Maxillofacial Surgery, Tagore Dental College & Hospital, Chennai, IND

**Keywords:** orbital floor, conchal cartilage, blow out fracture, reconstruction

## Abstract

According to Kim and Jeong (2016), isolated orbital fractures are encountered in 4-16% of all facial fractures, and orbital fractures composed approximately 30-­55% of the zygomaticomaxillary complex (ZMC) and naso-orbital-ethmoid (NOE) fractures. The ideal material for orbital floor fracture repair is one that is resorbable, osteoconductive, resistant to infection, minimally reactive, does not induce capsule formation, has a half-life that allows significant bone in-growth to occur, and is cheap and readily available.

In this article, we report a case of a young female with an orbital floor fracture managed surgically using conchal cartilage and a review of literature.

## Introduction

The orbit is a four-sided pyramidal structure that is weedy and exposed, especially the medial wall and floor. The floor is the shortest of all the walls, measuring 35-40 mm and terminating just before the orbital apex and the annulus of Zinn [1]. It is frequently fractured because of its delicate anatomy.

According to Ng et al., orbital floor fractures were first described in Paris by MacKenzie in 1844 [2]. The orbital floor shows the greatest degree of deformation in terms of static loading compared to the other orbital walls, which explains the high rate of floor fractures associated with blunt trauma. A 3-mm downward displacement of the entire floor results in an increase of about 1.5 cm (5%) in orbital volume and roughly 1.0-1.5 mm of enophthalmos [3].

The aim of surgical repair is to restore anatomical and functional sustainability in order to avoid the recurrence of herniation of the orbital contents, to revert the ocular movements and orbital volume, and to restore the aesthetic appearance of the face.

Early recognition and diagnosis are important factors in treating such conditions. A change in the content ratio (i.e., the relationship between the eyeball, musculature and orbital fat and the volume of the orbit) is considered the main originating mechanism of enophthalmos and diplopia. Alternatively, a minor cause, the atrophy of orbital contents, has also been reported as an aetiology of these alterations [4].

Pathophysiology

In 1889, Lang originally described this type of orbital fracture and termed it, traumatic diplopia or enophthalmos. Currently, it is commonly known as an orbital blowout fracture. Since its first description, various studies have assessed the mechanism of orbital floor fractures. In 1901, Lefort described that orbital fractures occur as a buckling mechanism secondary to forces applied to facial buttresses. Then, in 1943, Pfeiffer hypothesized a hydraulic mechanism in which force/pressure is directed onto the thin, fragile orbital bones [5].

Subsequently, many authors have studied and inveterated the fact that impact on the globe or inferior orbital rim can cause pure blowout fractures. Direct trauma to the globe (hydraulic mechanism) results in larger defects, involving both the orbital floor and medial wall, as well as significant herniation of the orbital contents. While each mechanism has been proven to occur in isolation, the clinical presentation of such injuries may result from some combination of the two [6].

Surgical approaches

Approaches to the repair of the orbital floor include transconjunctival, subciliary, mid-lower eyelid, infraorbital and endoscopic transantral [7-8]. The success of the surgical orbital floor reconstruction is determined by the postoperative restoration of the normal anatomical relations of the orbit and by avoiding complications of the surgical procedure and implants/materials used for reconstruction.

The graft materials can be divided into autologous grafts, allogenic materials and alloplastic materials. Conchal cartilage possesses characteristics and advantages similar to those of cartilage grafts, formerly used in orbital reconstruction [9]. Shaping via cartilage crusher delivers favourable results when necessary, hence, conchal cartilage is better adapted than septal cartilage, and its malleability is superior to bone grafts [10].

For this particular case, we opted for auricular cartilage because of its shape, which is very similar to that of the orbital floor, malleability, good functional support, limited morbidity of the donor site, the simplicity and speed with which it can be harvested, and, finally, the socio-economic status of the patient.

## Case presentation

A 23-year-old healthy female patient presented to the Outpatient Department (OPD), Department of Oral & Maxillofacial Surgery (OMFS), Tagore Dental College & Hospital with pain and swelling in the left side of the face following a road traffic accident which occurred eight days prior. She was primarily managed in a local hospital with antibiotics and analgesics before being referred to our hospital.

**Figure 1:**
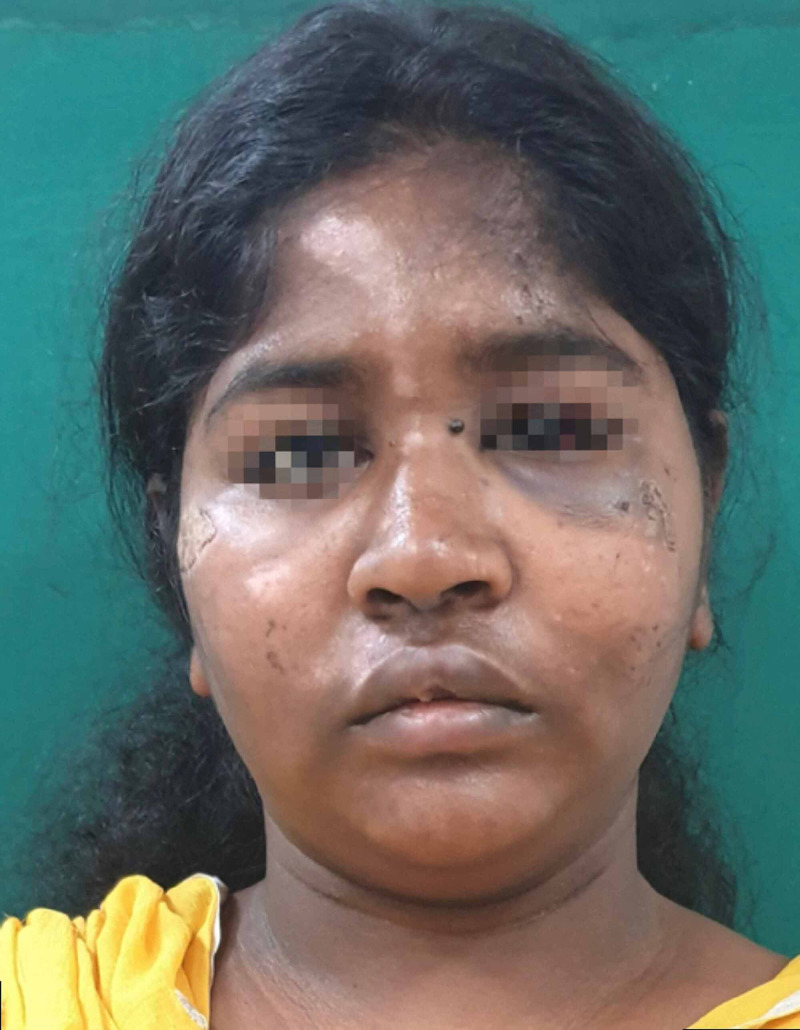
Pre-operative

On general examination, the patient was well built, conscious and oriented; her vital signs and all other systems were within normal limits. On extraoral examination, multiple abrasions and lacerations were observed on the left side of her face and right forehead. Significant left periorbital swelling with associated ecchymosis and petechiae was present. Her pupils were equal and reactive to light, and there was no evidence of enophthalmos, no pain with eye movement and no gaze limitations but subconjunctival haemorrhage involving the lateral aspect of the left eyeball was clearly noticeable. The patient was referred to an ophthalmologist and on ocular examination, her visual acuity and intraocular pressure (IOP) found to be normal. Tenderness over the left infraorbital margin and nasal bones was present (Figure 1).

**Figure 2:**
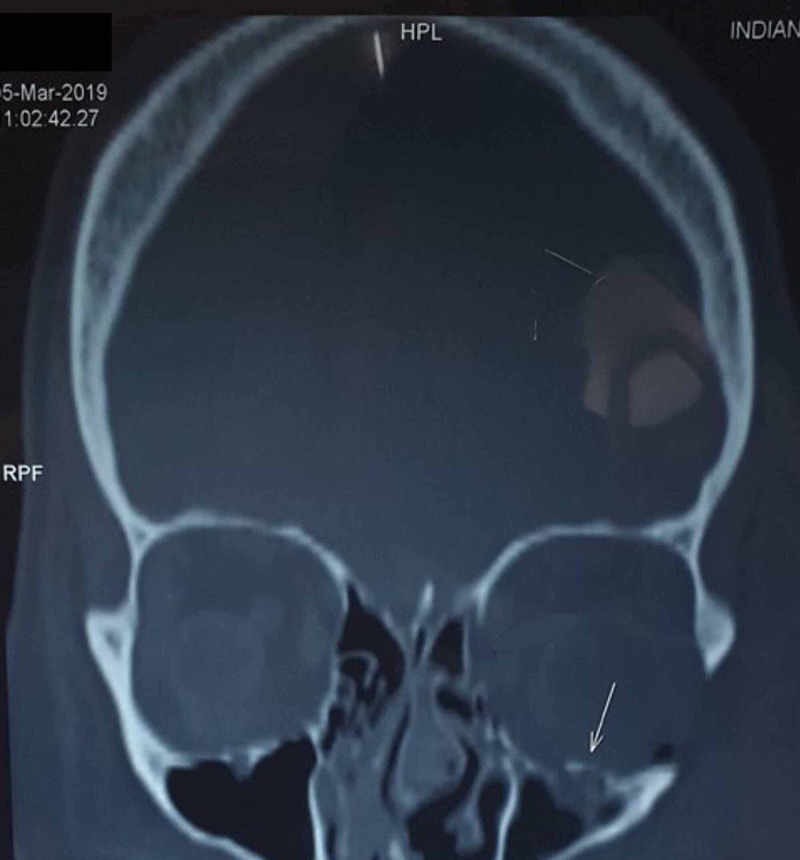
Preop CT showing left orbital floor fracture

Computerized tomography suggested a fracture to the inferior wall of the left orbit, which allowed the herniation/protrusion of infraorbital content into the left maxillary sinus (Figure 2). Accordingly, we planned to reconstruct the inferior wall of the left orbit using the right conchal cartilage.

Surgical technique

Recipient Site

**Figure 3:**
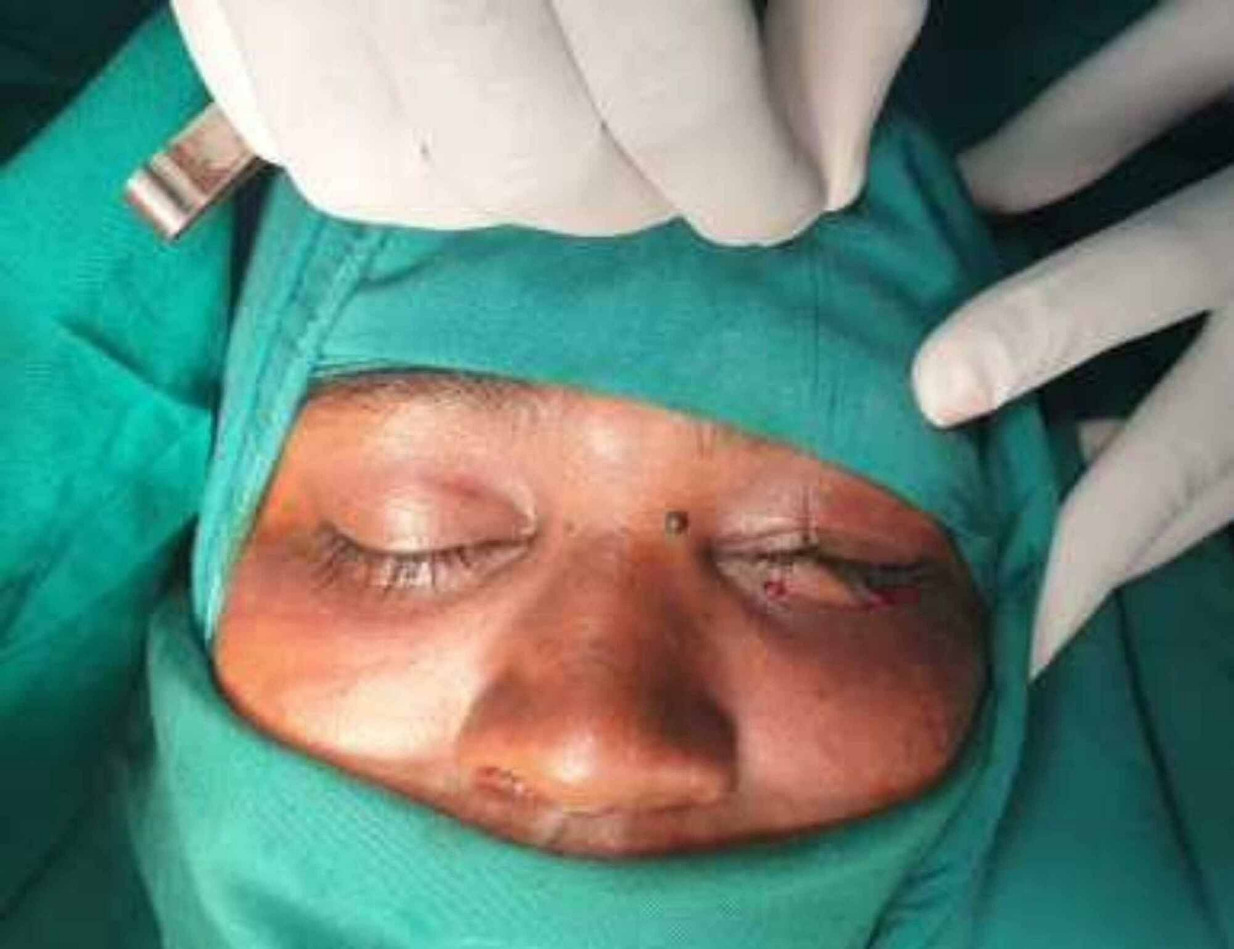
Traction suture using 5-0 silk

After infiltration of the inferior orbital rim with 2% lignocaine with 1:80,000 adrenaline, a traction suture was done for retraction. Ideally, a subtarsal approach is taken along the lower border of the tarsal plate in the subtarsal fold, but in this case, since the subtarsal fold was obscured by oedema, we made the incision 6 mm below the lower eyelid margin (Figure 3).

The orbicularis oculi muscle was then identified and blunt dissection was done in the direction of its fibres down to a few millimetres below the skin incision to avoid scar inversion and preserve the innervation of the orbicularis oculi muscle [11].

**Figure 4:**
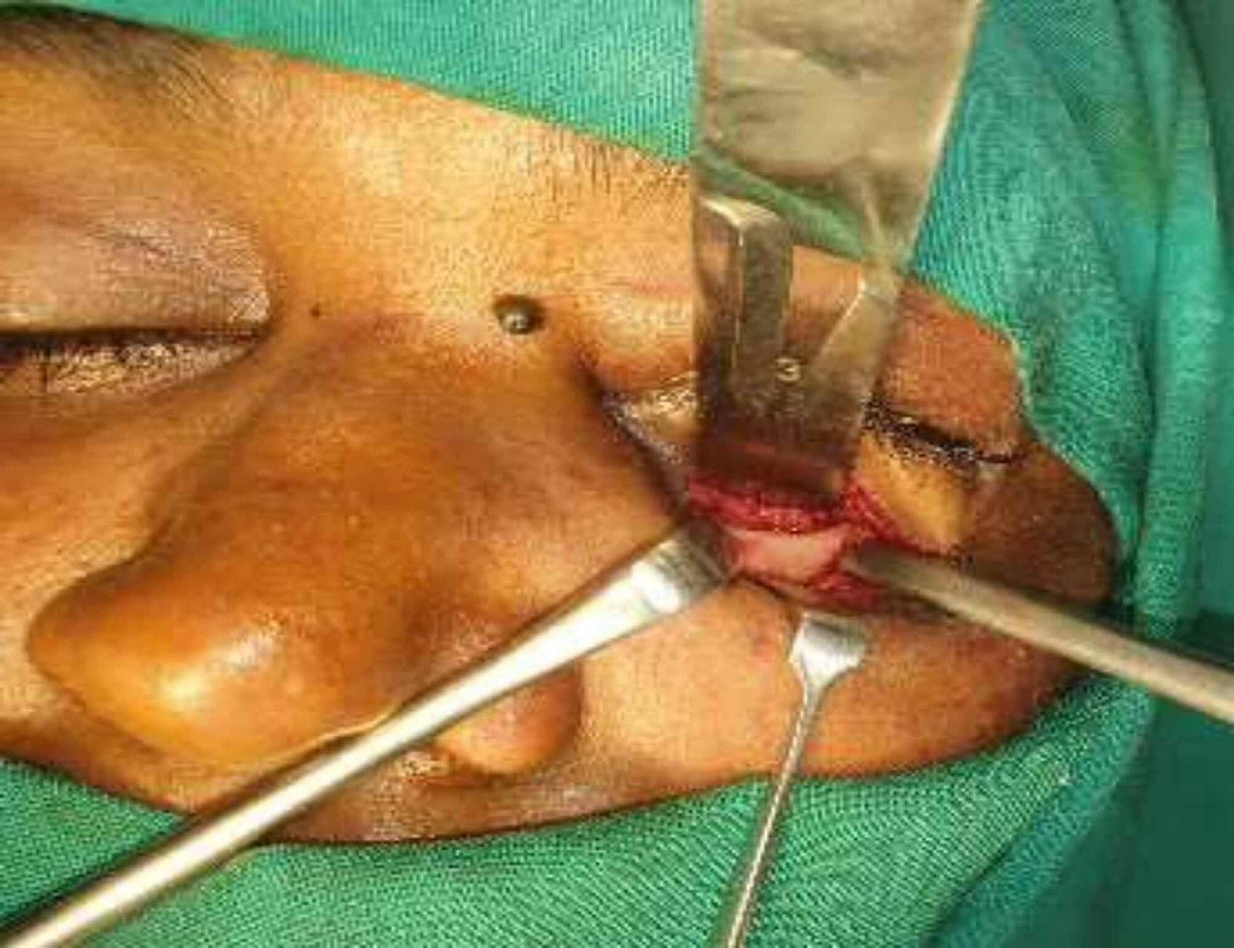
Fracture site exposed

The incision was then carried down to the level of the infraorbital rim in the preseptal plane. Then, the periosteum was incised, blunt dissection was performed, the fracture was exposed, herniated soft tissues were teased out from the maxillary sinus to the orbital cavity (Figure 4) and size of the defect was measured as 1.5 x 1 cm using a template assessed from the CT scan.

Donor Site

**Figure 5:**
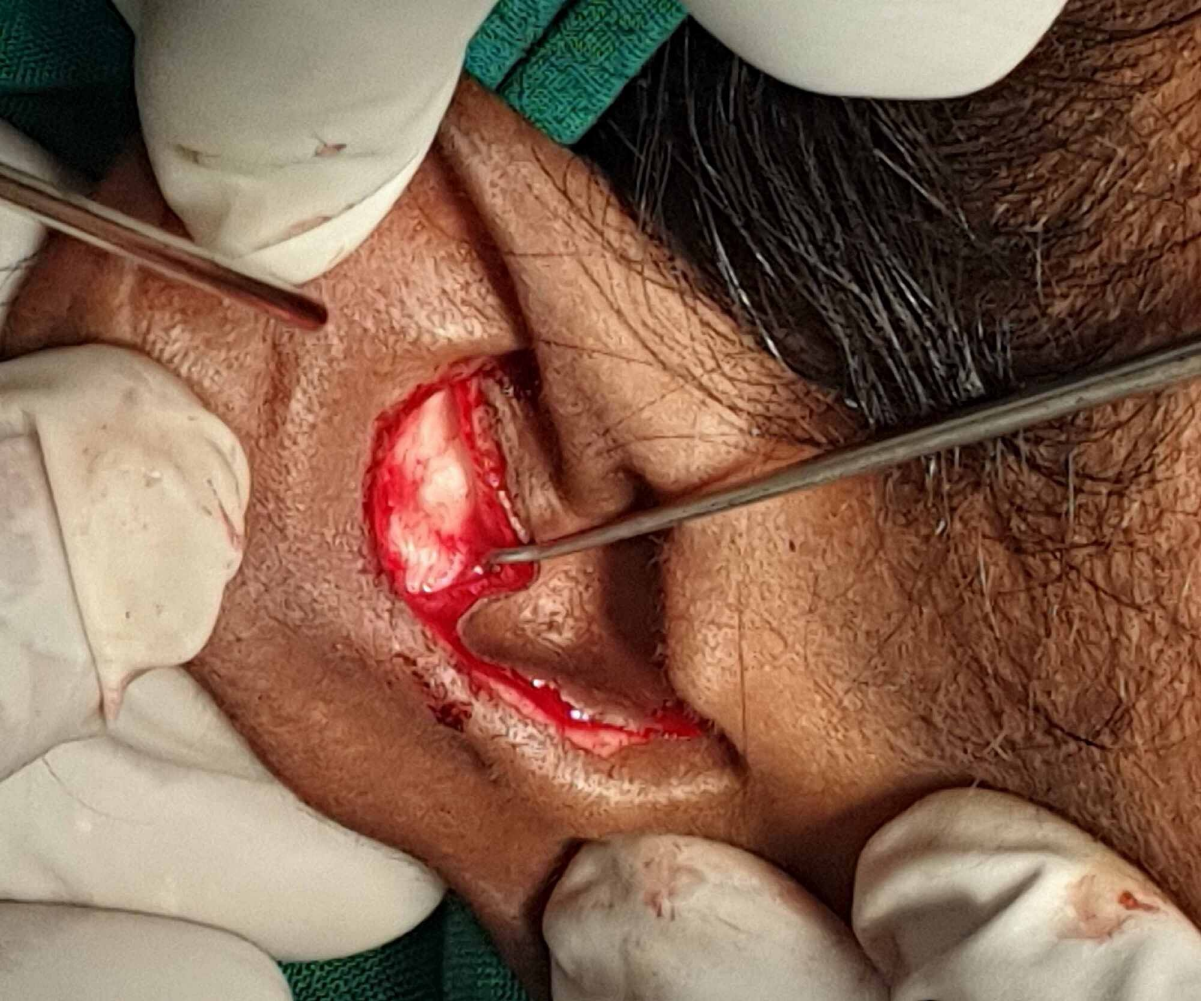
Conchal bowl incision in the right ear

**Figure 6:**
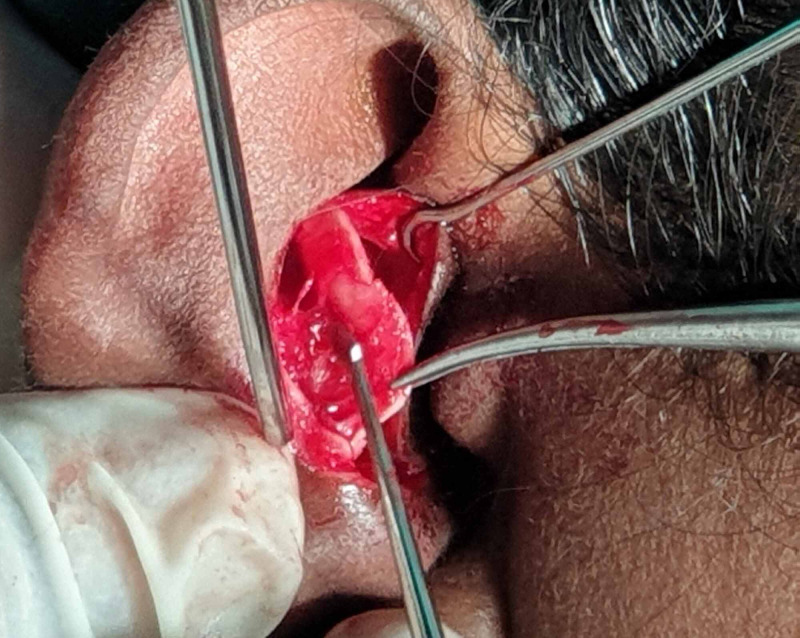
Sharp dissection of the perichondrium

**Figure 7:**
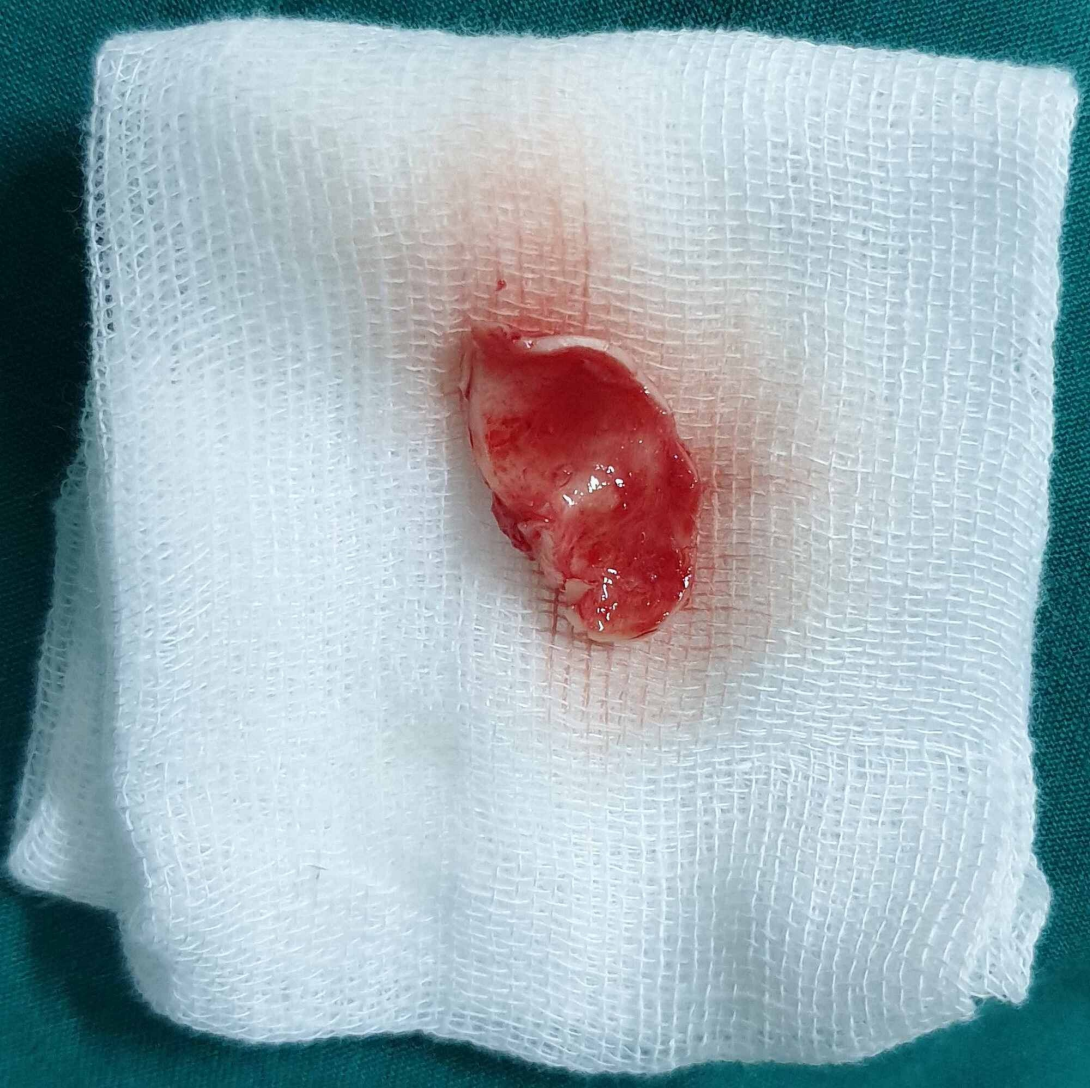
Harvested conchal cartilage

Subcutaneous injection of 2% lignocaine with 1:80,000 adrenaline was injected along the conchal bowl of the right ear. No 15-bard parker blade was used to make an incision just inside the helical bowl, leaving a 2 to 3 mm margin of an outer rim of the conchal bowl. Then the incision was carried down through the perichondrial layers, and the anterior perichondrium was then sharply dissected from the conchal cartilage. Based on the defect size (1 x 1.5 cm) which was assessed earlier, the conchal cartilage was harvested of size 2 x 2 cm with the posterior perichondrium intact using an intraoperative template (Figures 5-7). The incision was closed using 6-0 ethilon, and a tie-over bolster dressing was placed, over which the gauze dressing was secured using Elastoplast.

Graft in Position

**Figure 8:**
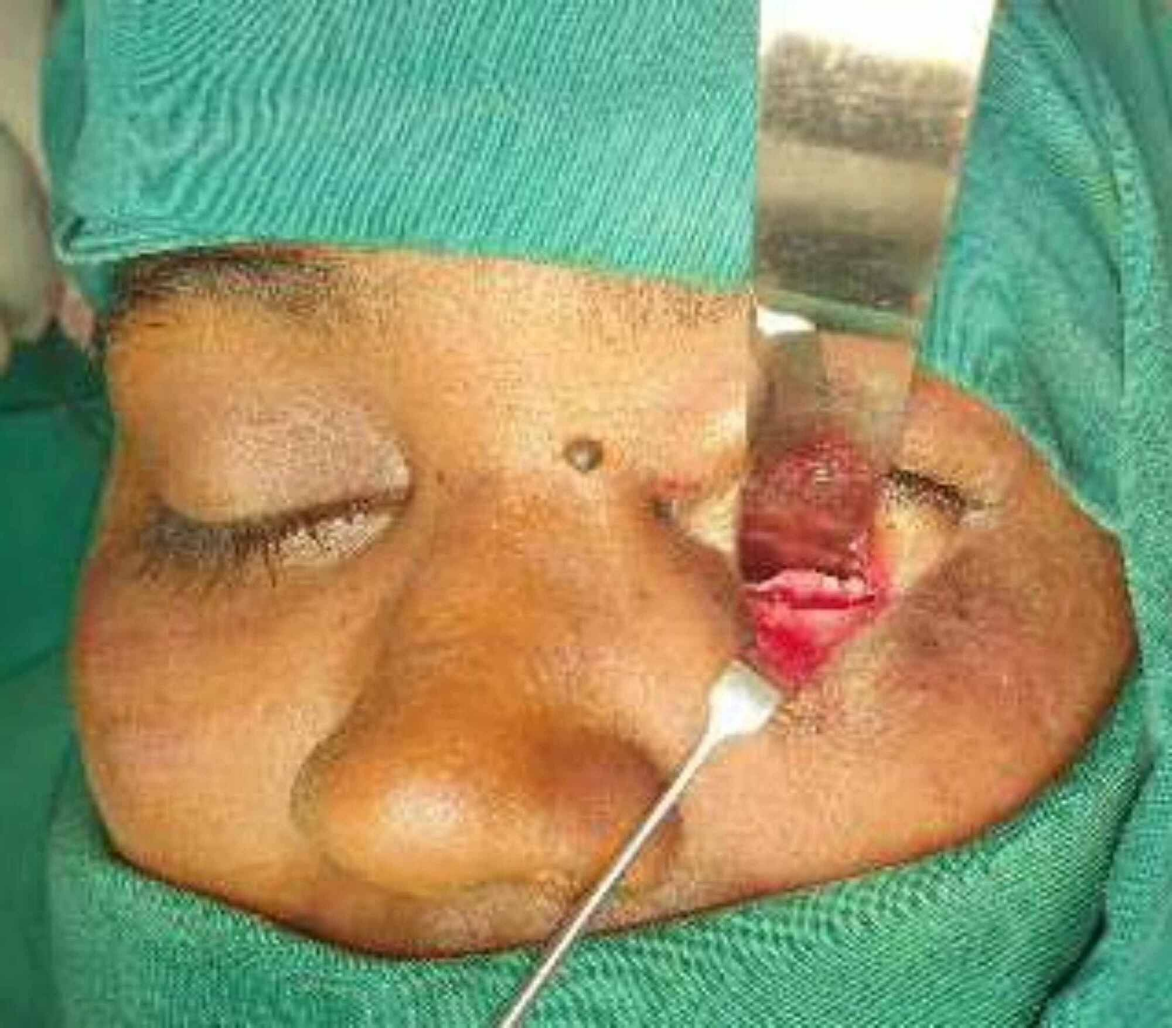
Conchal cartilage in left floor of the orbit

After teasing out the herniated orbital fat from the maxillary sinus, the obtained conchal cartilage was adapted in the orbital floor defect area and posteriorly the graft was seated on the sound bone. The thickness of the cartilage and its concave shape facilitated counter-sink of the graft to the concave orbital floor. So, there was no requirement for the sutures. Subsequently, the incision was closed in layers using 3-0 vicryl and 5-0 ethilon (Figure 8).

**Figure 9:**
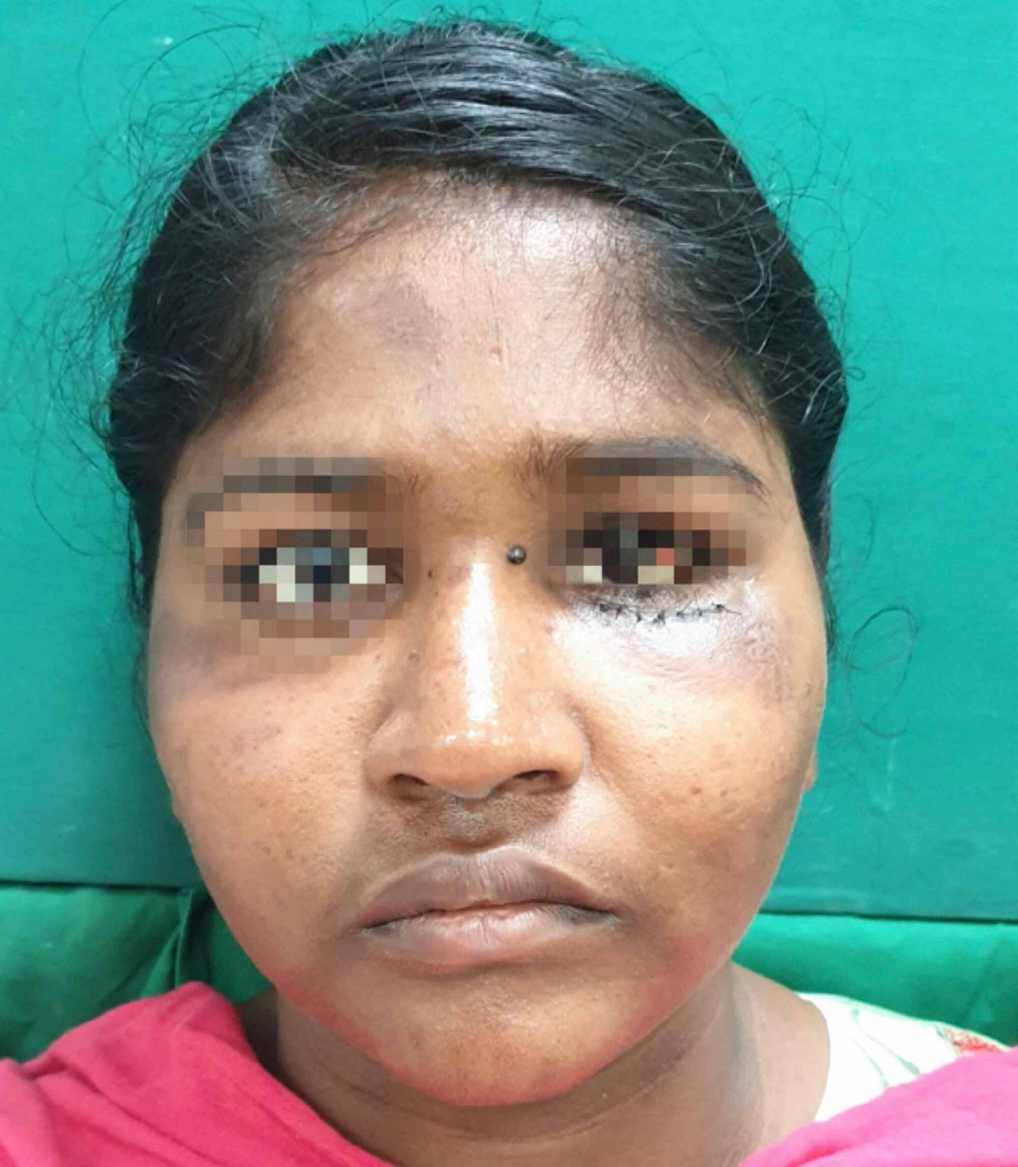
Postop - 1 week recipient site

**Figure 10:**
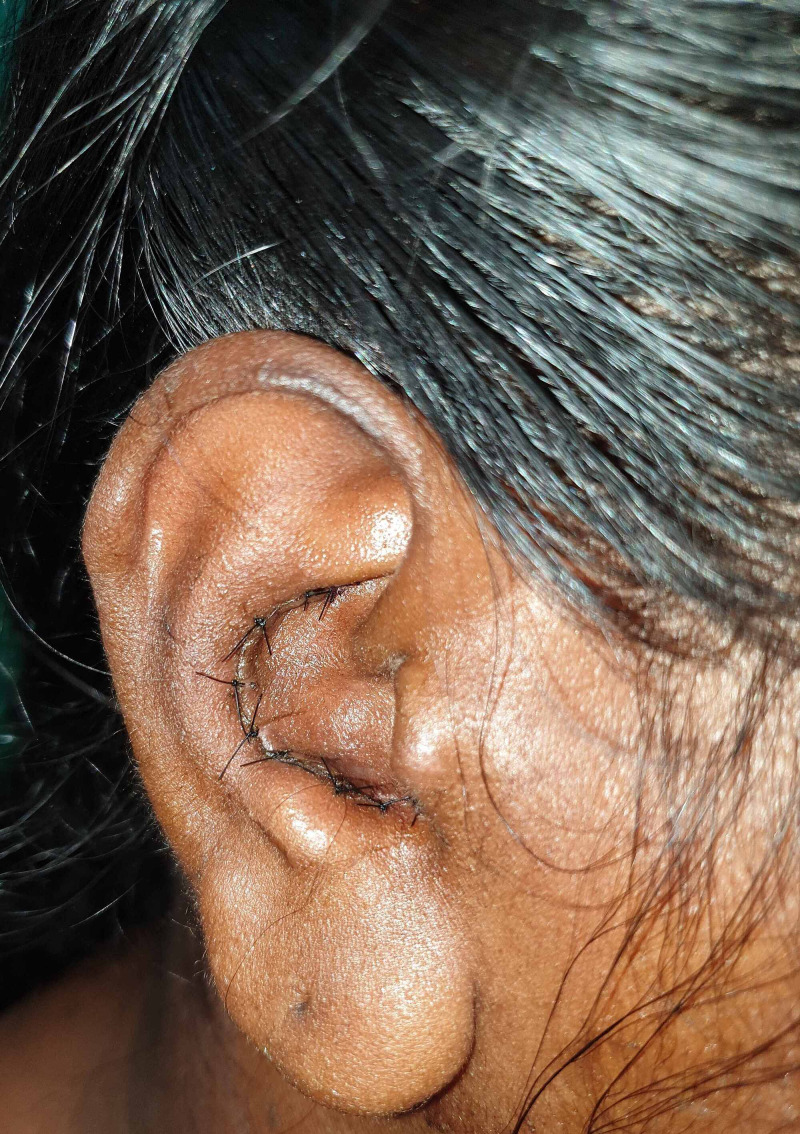
Postop - 1 week donor site

**Figure 11:**
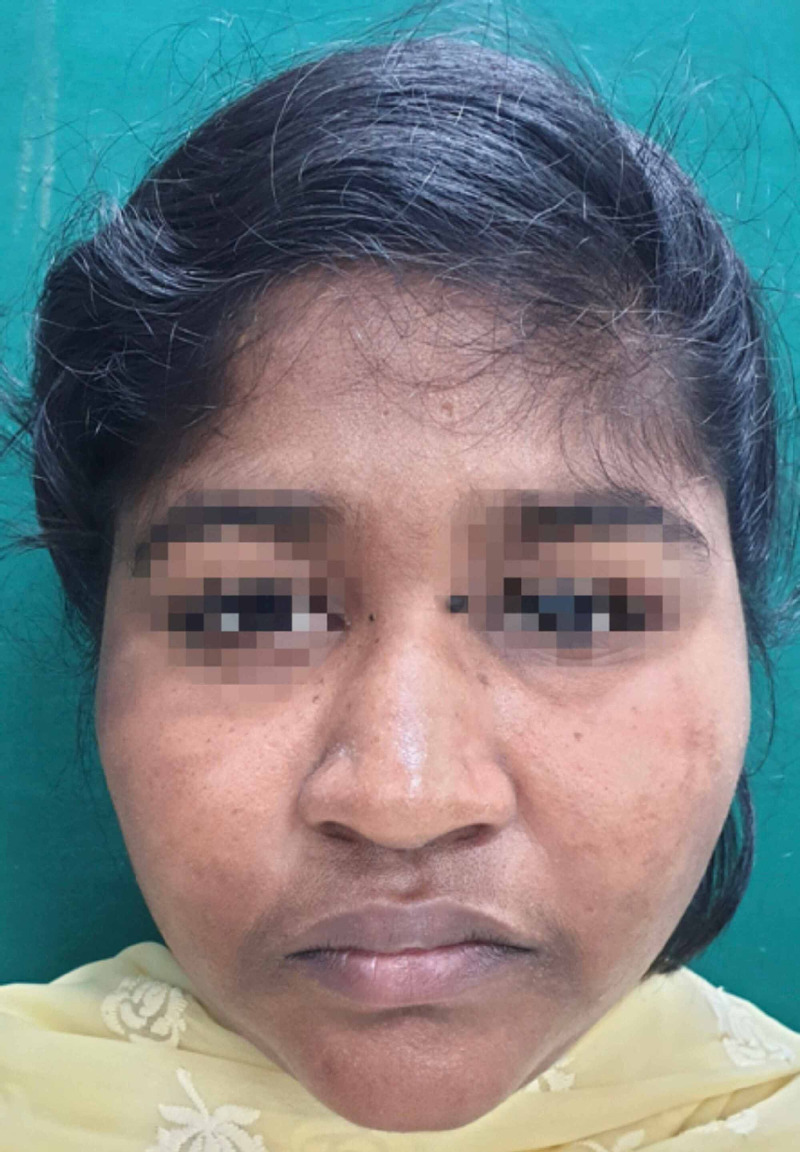
Postop - 6-month recipient site

**Figure 12:**
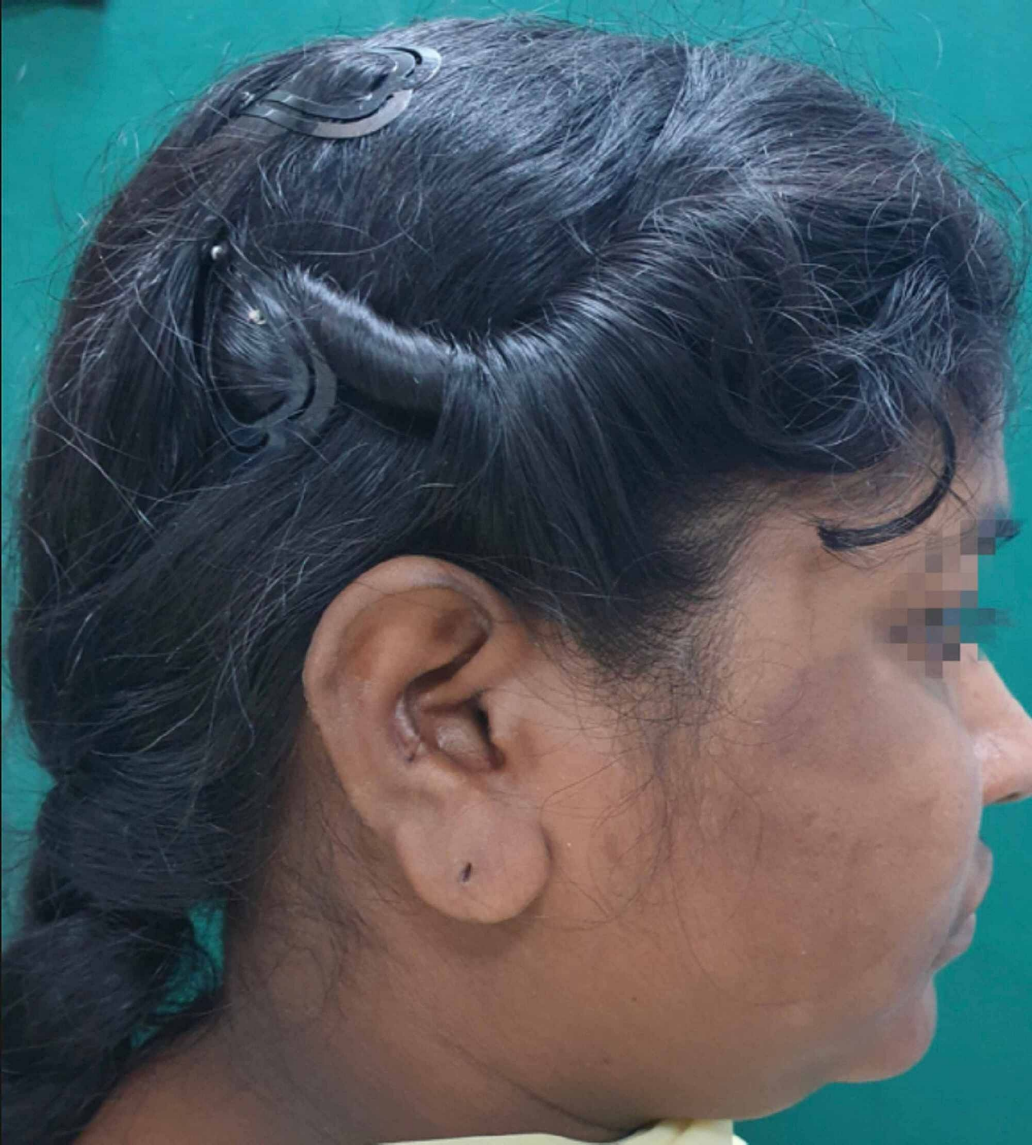
Postop - 6-month donor site

**Figure 13:**
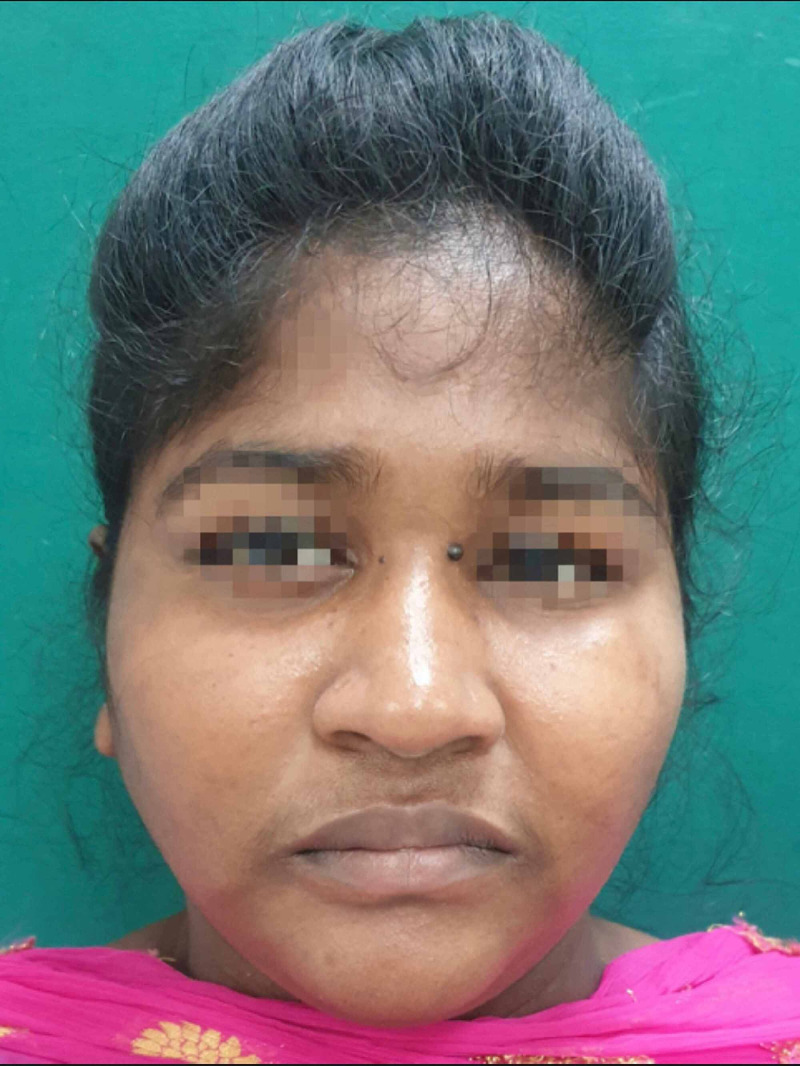
Postop - 1 year recipient site

**Figure 14:**
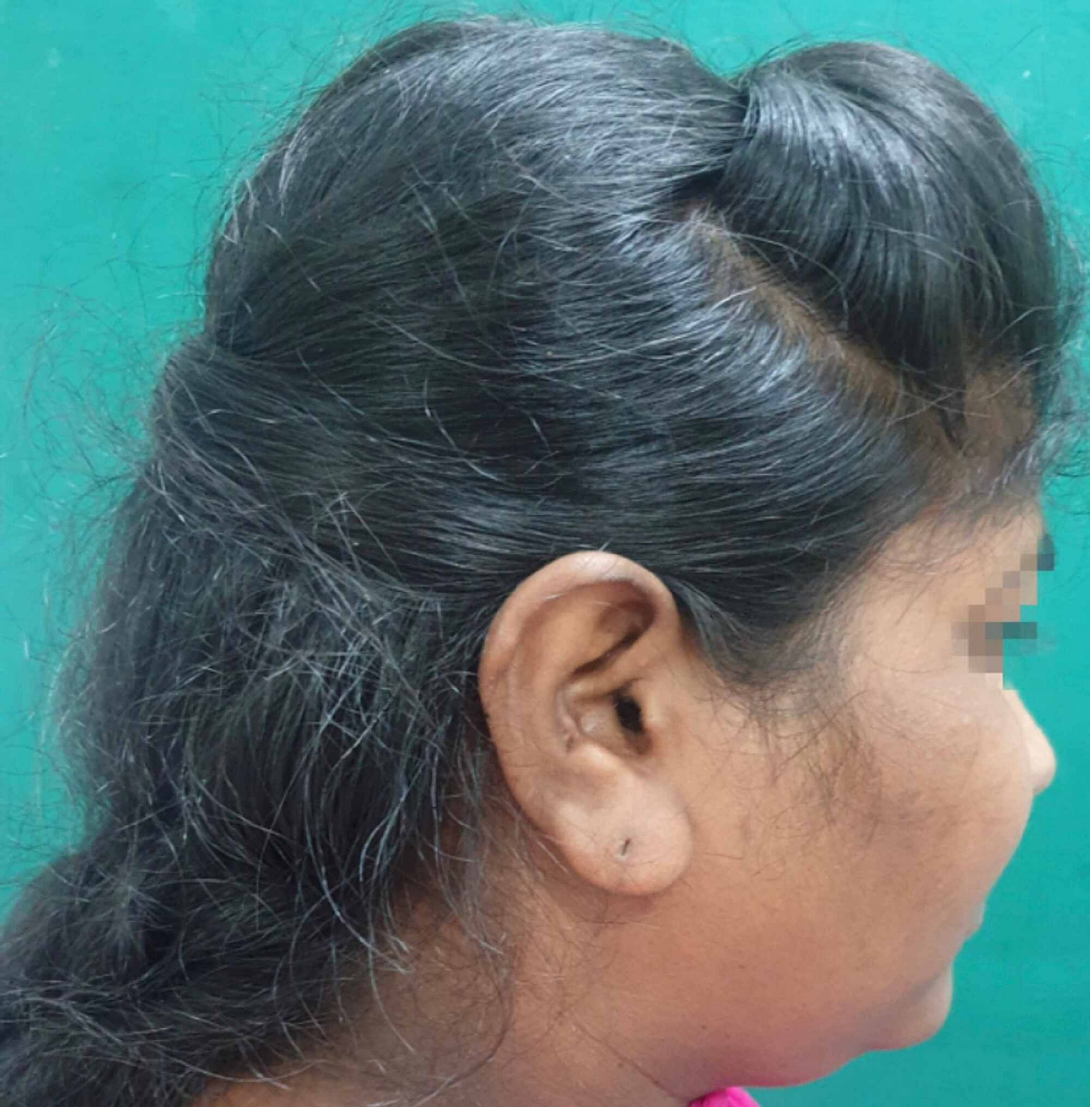
Postop - 12-month donor site

**Figure 15:**
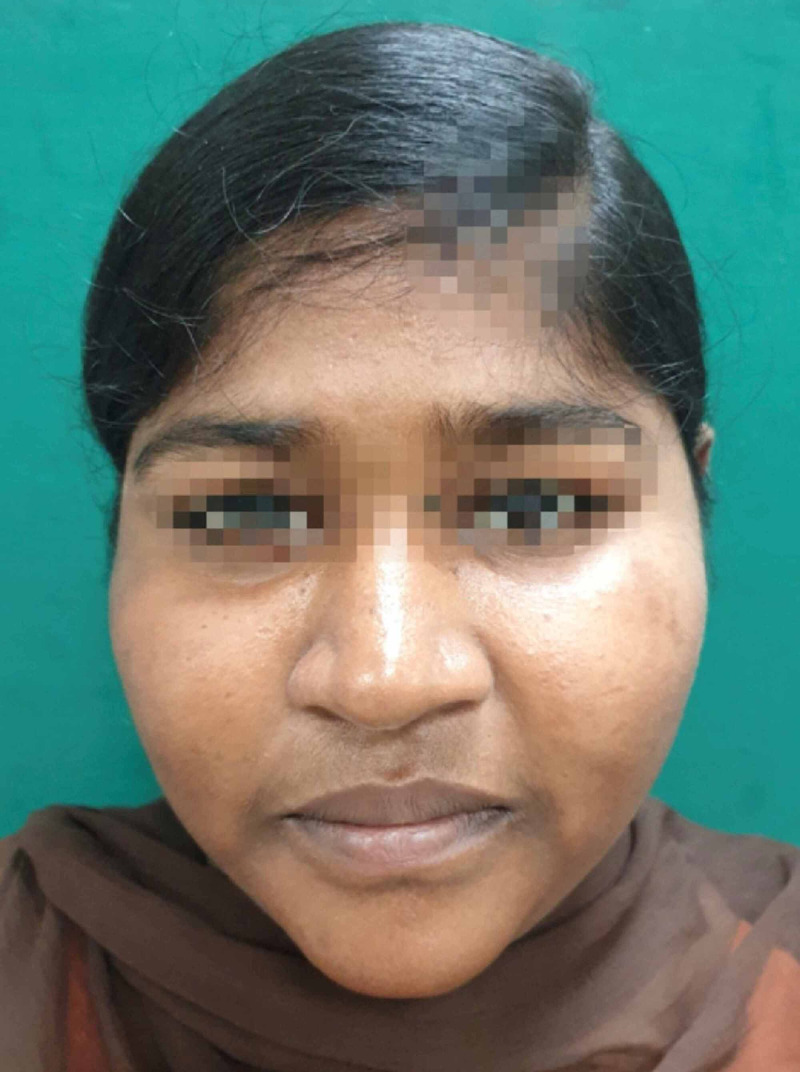
Postop - 22-month recipient site

**Figure 16:**
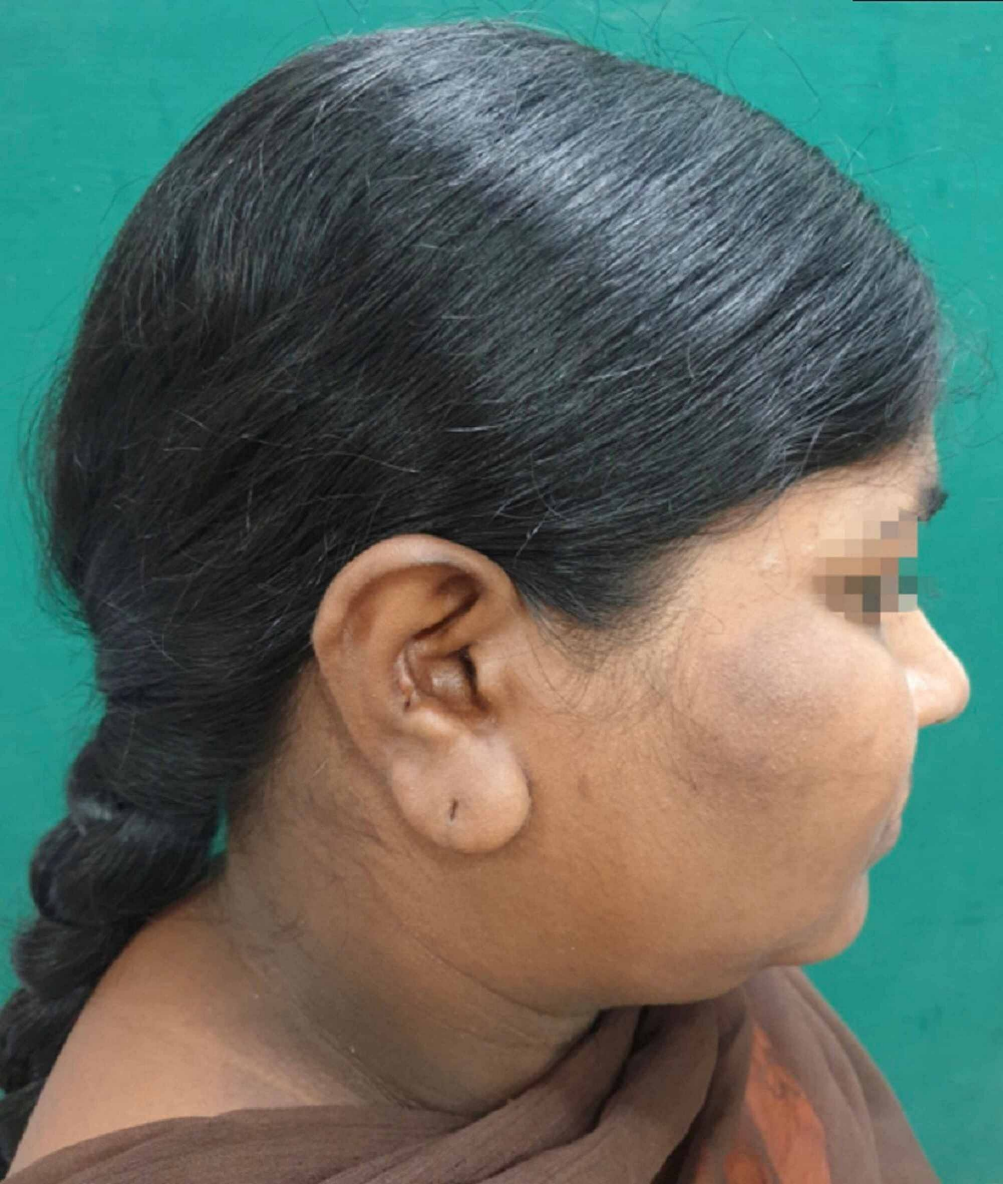
Postop - 22-month donor site

**Figure 17:**
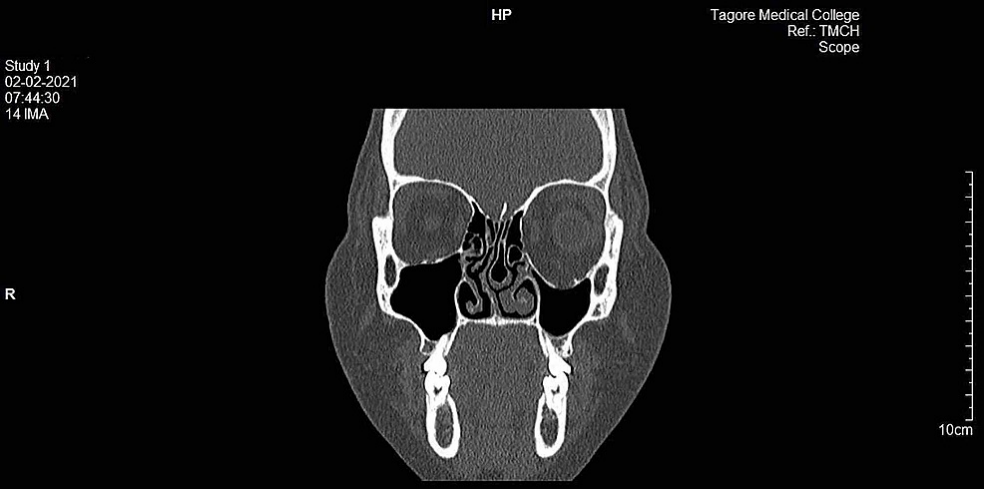
Postop 22-month CT scan (coronal): The orbital contents repositioned within the orbit and the floor has been reconstructed with cartilage graft

**Figure 18:**
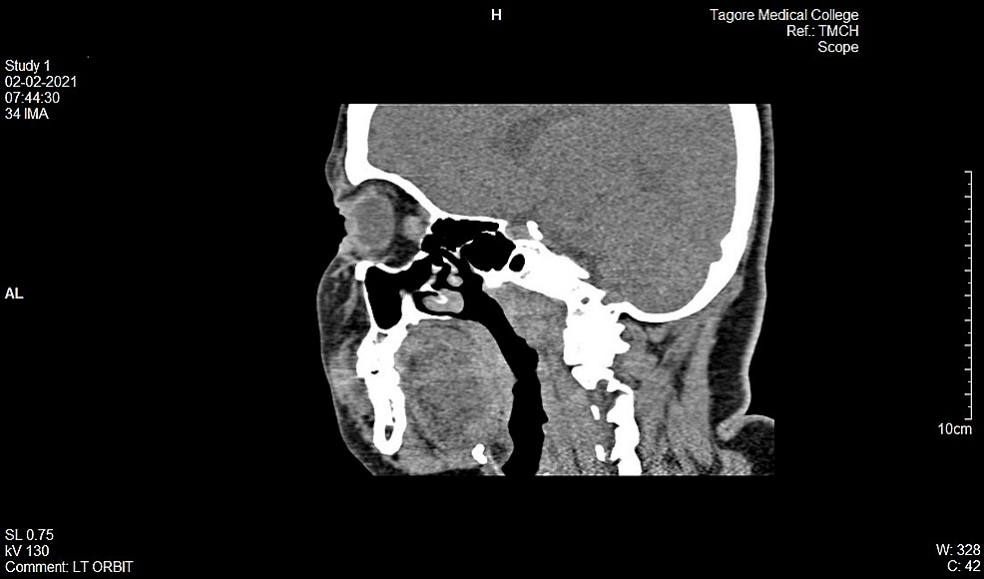
Postop 22-month CT scan (sagittal): Posteriorly the graft seated in a sound bone

**Figure 19:**
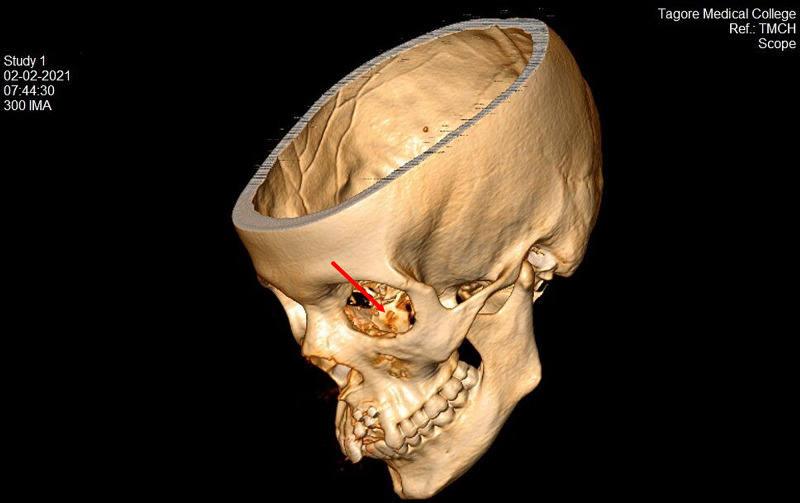
Postop 22-month CT scan (3D) view showing the graft in position (red arrow) in the left orbit

Postoperatively the donor site was clinically assessed and the healing was satisfactory without any morbidity. The recipient site follow-up was done both clinically and radiographically. Clinically, there was no signs of infection in the operated site and also a significant improvement in the subconjunctival haemorrhage in the left orbit. Computed tomography (CT) scan was done after 22 months to confirm the proper positioning of the soft tissues within the orbit (Figures 9-19). However, few immediate postop sequelae like pain and swelling were observed which settled subsequently.

## Discussion

According to Laskin and Sarnat, cartilage is minimally vascularized and thus requires little blood perfusion, which means that it undergoes less resorption than bone grafts. Although cartilages are predominantly avascular, the perichondrium is the main source of blood supply [12]. Nearly 50 years ago, in 1969, Stark and Frileck stated that conchae are reservoirs of autogenous cartilage, leaving no cosmetic or functional deformity. Its shape is well-designed to furnish suitable grafts for smaller saddle deformities in the nose and orbital floor defects and act as a reinforcing strut of helical crus to prevent warping [13]. Constantian, in 1981, also mentioned that autogenous tissues had proven their superiority over inorganic implants in many reconstructive surgeries. He stressed that using cartilage in orbital floor reconstruction is much better than other autogenous grafts because it is easy to obtain and shape and lacks evidence of resorption in long-term follow-up [14]. In 1992, Hendler et al. emphasized that auricular cartilage is an excellent source of autogenous tissue appropriate for the repair of orbital floor defects. The thickness of this cartilage and its concave shape are ideal qualities that enable a precise fitting to the orbit’s concave floor, especially at the junction of the floor with the medial wall, the most common location of disruptions caused by trauma.

The intrinsic characteristics of this cartilage make it highly suitable for the reconstruction of large defects. The long-term survival of fresh cartilage autografts has been demonstrated experimentally, and clinical investigation has proved that fresh cartilage autografts maintain adequate structure and volume years after transplantation. The added advantage of using auricular cartilage is that donor and recipient sites can be merged into a singular surgical field due to their close proximity. This confers ease to the preparation and site-draping processes; the cartilage can usually be obtained in less than 20 minutes via simplistic surgical technique, adding minimal time to the procedure [15].

In 2002, Castellani et al. discussed a follow-up of 14 cases in which the author compared the autogenous grafts with biomaterials and emphasized the limitations of the autografts, including a) risk of resorption, especially in the posterior third of the orbit; b) increased risk of graft displacement; c) difficulty in remodelling the grafts; d) donor site morbidity; e) prolonged operative duration [16]. What is more, Mok et al., mentioned that autogenous tissues were among the first materials used to reconstruct the internal orbit, and they are too flexible to provide adequate support for the orbital contents when used in large defects [9]. The most critical arguments against the use of autogenous materials, particularly, is regarding autogenous bone grafts. To a lesser degree, cartilage can also undergo unpredictable resorption, leading late enophthalmos [8].

In 2009, Bayat et al. compared autogenous septal nasal cartilage and conchal cartilage as grafts for reconstruction of orbital blowout fractures. Though nasal septal grafts and auricular grafts have both been recommended for the reconstruction of blowout fractures, the authors were unaware of any study that had shown that one graft results in better outcomes in the orbital reconstruction of blowout fractures than the other [17].

Saluja et al. discussed the characteristics of cartilage, including its low anaerobic metabolism and relative vascularity, which allows cartilage grafts to survive with minimal requirement for oxygen/perfusion, thereby improving the graft viability and reducing the resorption rates when compared with bone grafts [18].

Critics, however, have argued that conchal cartilage is too flexible and does not provide enough support for the orbital contents. Gunarajah and Samman conducted a systematic review to evaluate the reported use and outcomes of implant materials utilized for the restoration of post-traumatic orbital floor defects in adults. The authors stressed that autogenous cartilage is a durable and resilient material that is still malleable, rendering it extremely pliable to the orbital walls. It also has a reduced rate of resorption when compared with a bone; hence, it demonstrated a period of longer viability and structural integrity, as shown in animal studies [19].

In 2014, Gart and Gosain demonstrated that deficient treatment might lead to complications such as diplopia, orbital dystonia, unsightly appearance, exophthalmos, and restrictions of ocular movement caused by the entrapment of intraorbital fat or extraocular muscles within fracture fragments or reconstruction material [20].

## Conclusions

Autogenous grafts are better than alloplastic materials because of its biocompatibility. Conchal cartilage because of its morphologic characteristics facilitates better adaptation with acceptable rigidity, less donor site morbidity and low infection risks of the recipient site. In our case on follow-up after 22 months, we observed no morbidity of the donor area and the recipient site as well. Conchal cartilage is definitely an alternative to other orbital floor reconstruction materials, especially for the smaller defects.
